# Transparency in Cognitive Training Meta-analyses: A Meta-review

**DOI:** 10.1007/s11065-024-09638-2

**Published:** 2024-04-19

**Authors:** Alejandro Sandoval-Lentisco, Rubén López-Nicolás, Miriam Tortajada, José Antonio López-López, Julio Sánchez-Meca

**Affiliations:** Department Basic Psychology and Methodology, University of Murcia, Murcia, Spain

**Keywords:** Cognitive training, Meta-review, Transparency, Practices, Eligibility criteria

## Abstract

Meta-analyses often present flexibility regarding their inclusion criteria, outcomes of interest, statistical analyses, and assessments of the primary studies. For this reason, it is necessary to transparently report all the information that could impact the results. In this meta-review, we aimed to assess the transparency of meta-analyses that examined the benefits of cognitive training, given the ongoing controversy that exists in this field. Ninety-seven meta-analytic reviews were included, which examined a wide range of populations with different clinical conditions and ages. Regarding the reporting, information about the search of the studies, screening procedure, or data collection was detailed by most reviews. However, authors usually failed to report other aspects such as the specific meta-analytic parameters, the formula used to compute the effect sizes, or the data from primary studies that were used to compute the effect sizes. Although some of these practices have improved over the years, others remained the same. Moreover, examining the eligibility criteria of the reviews revealed a great heterogeneity in aspects such as the training duration, age cut-offs, or study designs that were considered. Preregistered meta-analyses often specified poorly how they would deal with the multiplicity of data or assess publication bias in their protocols, and some contained non-disclosed deviations in their eligibility criteria or outcomes of interests. The findings shown here, although they do not question the benefits of cognitive training, illustrate important aspects that future reviews must consider.

In the last years, there has been growing concern regarding the transparency, reproducibility, and replicability of psychological science. Failed large-scale attempts to replicate empirical studies (Open Science Collaboration, 2015; Shrout & Rodgers, 2018) have harmed the credibility of the research conducted in the field (Baker, 2016).

Carrying out a meta-analysis, just like primary studies, involves making decisions at different stages, such as the design of the study, data collection, data analysis, and reporting of the results. To illustrate the myriad decisions that have to be made when carrying out a meta-analysis, we have created a non-exhaustive scheme (see the Appendix). This particular scheme is intended for the cognitive training field but can be easily adapted to most meta-analyses.

Importantly, many of these decisions are often rather arbitrary, as they lack a strong methodological or substantive rationale. This element of subjectivity is often referred to as “researcher degrees of freedom” (Hardwicke & Wagenmakers, 2021; Simmons et al., 2011; Wicherts et al., 2016). Acknowledging the researcher degrees of freedom in meta-analysis is important as well because they can have an impact on the results (Ioannidis, 2016; Maassen et al., 2020; Page et al., 2013).

Considering the relevant role that meta-analysis has in science, it is of great importance that every meta-analytic decision that could influence the results or conclusions is transparently reported, so that anyone can evaluate the methodology and the potential for bias (Ioannidis, 2005; Kvarven et al., 2020; Lakens et al., 2017). This way, if we had all the information about each decision and procedure and, additionally, the authors made their data available too, this would strongly increase the chances that the results are *reproducible*, i.e., we would obtain the same results by applying the methods that are reported by the authors to their data (Epskamp, 2019; López-Nicolás et al., 2022), and this would also enable other authors to conduct re-analyses with novel or alternative meta-analysis methods.

Previous attempts to inspect the reproducibility of meta-analyses have found problems because the articles were often missing important methodological aspects and the necessary data (Lakens et al., 2017; Maassen et al., 2020). Recently, Polanin et al. (2020) and López-Nicolás et al. (2022) have examined the transparency and reproducibility-related reporting practices in different samples of psychological meta-analyses. In particular, Polanin et al. (2020) selected meta-analyses published in the journal *Psychological Bulletin*, while López-Nicolás et al. (2022) randomly selected meta-analyses from the field of clinical psychology. They both found important issues regarding the reporting of the necessary information to reproduce the search of studies, lack of detail on the exact formula used for the summary statistics, lack of availability of essential data from primary studies such as means and standard deviations, and failure to report the script code used. Besides, they found modest rates of appropriate reporting of other important information such as the effect sizes of primary studies, their funding sources, and possible conflicts of interest. Equally important, most of the data from primary studies that was available was not in a machine-readable format; i.e., it was not in a format that could be easily read and manipulated for being reused (Wilkinson et al., 2016), which greatly hinders attempts to check the reproducibility or make any further re-analysis of the data.

For this meta-review, i.e., an overview of systematic reviews (López-López et al., 2022), we aimed to inspect these transparency practices in meta-analyses that assessed the benefits of cognitive training. The term cognitive training refers to the repeated practice of specialized tasks that target specific cognitive functions, such as memory or executive functions (Clare & Woods, 2004; Huntley et al., 2015). It is assumed that this kind of training can result in an improvement of the functioning of the trained cognitive domain and, potentially, a transfer to other tasks and abilities beyond the trained context, including daily life (Huntley et al., 2015; Redick, 2019). Remarkably, cognitive training is currently being used as a treatment for different clinical conditions or as a “cognitive enhancement tool” delivered under commercial programs such as Cogmed, Lumosity, or Cognifit, to name a few (Simons et al., 2016).

Nonetheless, this research area has been controversial (Gobet & Sala, 2022; Harvey et al., 2018; Sala & Gobet, 2019; Simons et al., 2016; Traut et al., 2021), and it has been crowded with contradictory results, not only between primary studies (Simons et al., 2016) but also between meta-analyses. As a notable example, Au et al. (2015) and Karbach and Verhaeghen (2014) published meta-analyses that evidenced the benefits of working memory training. Later on, Melby-Lervåg and Hulme (2016) re-analyzed the data from these two meta-analyses, reaching less optimistic conclusions. This re-analysis also received a subsequent reply from Au et al. (2016) where they reaffirmed their original conclusions. Although working memory training is probably the most popular and polemical area (Aksayli et al., 2019; Hou et al., 2020; Nguyen et al., 2019; Redick, 2019; Teixeira-Santos et al., 2019), there are some other examples of cognitive training meta-analyses with conflicting results. When examining the evidence of cognitive training in healthy older adults, meta-analyses varied importantly in their conclusions (Jones et al., 2021; Lampit et al., 2014; Nguyen et al., 2022). Similarly, meta-analyses whose target was children did not seem to agree either (Karch et al., 2013; Kassai et al., 2019; Sala & Gobet, 2017, 2020; Scionti et al., 2020). Last, it is worth adding that Gavelin et al. (2020) conducted a meta-review including systematic reviews and meta-analyses of cognitive training and other cognition-oriented treatments, such as cognitive rehabilitation or cognitive stimulation specifically in older adults (see Clare & Woods, 2004; Gavelin et al., 2020 for a clarification of the terms). Here, authors found a small but significant effect for cognitive training, and interestingly, although the quality of reviews, as rated with the AMSTAR, was generally low, they also found a positive correlation between the quality of the review and the magnitude of the pooled effect size.

Thus, given the state of this research field, it is especially important that cognitive training meta-analyses transparently report all the information about the decisions that could influence any result or conclusion. This would help minimize the risk of bias arising from the authors of the meta-analyses as well as allow anyone to properly evaluate the methodological adequacy of already published meta-analytical studies.

## Purpose

The main aim of this study was to examine the transparency of the reporting of information in meta-analyses that examined the benefits of cognitive training programs. In addition, our results are compared to previous studies, checking whether transparency practices are similar across different disciplines. Moreover, in contrast to previous research assessing transparency practices in meta-analyses, this review also intends to serve as an illustration of the different meta-analytical practices in the field of cognitive training, showing the methods and decisions that authors have used for meta-analytical decisions such as defining the eligibility criteria.

## Method

### Inclusion of Studies

Our initial aim was to include systematic reviews reporting at least one original meta-analysis that assessed the benefits of cognitive training delivered to any population, clinical or not, of any age and under any format, computerized or not, excluding reviews mainly focused on non-cognitive training videogames, physical exercise, exergames, non-invasive brain stimulation, or Brain Computer Interface, even if they were applied in combination with cognitive training. The reason to exclude these studies was to focus on studies reviewing solely the effect of cognitive training, reducing heterogeneity between studies and making them more comparable. Nonetheless, we found several reviews that, although only labeled as cognitive training meta-analyses, also included some primary studies that delivered other interventions in combination with cognitive training or that used non-cognitive training videogames, such as real-time strategy or action videogames (Lampit et al., 2014; Oldrati et al., 2020). In order to examine a larger sample, we decided to include these reviews as well. Besides, we also included meta-analytic studies that were labeled as cognitive rehabilitation or remediation, as long as the majority (i.e., > 50%) of the primary studies delivered a cognitive training intervention. To check whether cognitive training studies were the majority in a meta-analysis, we individually checked the details provided by the meta-analytic studies for each of the primary studies that were included. We did not impose any further restrictions on publication year. We included reviews written in English or Spanish. Lastly, we decided to exclude network meta-analyses and Bayesian meta-analyses because these meta-analytic frameworks have their own specific procedures and evaluating the reporting of information of those specific methods would have entailed a large cost, requiring including many other items.

### Searching and Screening of Studies

We aimed to identify meta-analyses conducting a systematic electronic search in PubMed, Scopus, and all the collections of Web of Science. The search was carried out through February 2021 and updated in July 2023. A full search strategy for each database is available in the Supplementary Table 1 at https://osf.io/j58ax. No search limits were set. All references identified through the electronic searches were imported to Zotero. De-duplication was performed using the tool included in the software and manually revised. We first evaluated the title and abstract of each article for potential inclusion. Where inclusion or exclusion of a study was not clear from the information available in the title or abstract, we assessed the full text. Additionally, we screened the references of the articles that met the inclusion criteria. Screening was performed by one author (ASL). Doubts were discussed with two authors (JSM and JLL). A PRISMA 2020 flow diagram (Page et al., 2021) summarizing the search and screening process is available in the Supplementary Fig. 1 at https://osf.io/mp8ye/.

### Data Collection

A structured coding form containing items that covered different meta-analytical decisions was produced. This form was based on previous studies with similar scope (Hardwicke et al., 2020; Koffel & Rethlefsen, 2016; López-Nicolás et al., 2022; Pigott & Polanin, 2020; Wallach et al., 2018) and the PRISMA 2020 guidelines (Page et al., 2021). We preferred to use this approach over using the AMSTAR because the latter aims to evaluate quality of the review. Instead, we focus on the reporting of information of each decision, without assessing whether meta-analytic decisions were the most appropriate. Our approach also allowed us to evaluate a wider range of aspects such as data sharing, reporting of specific meta-analysis parameters, how authors performed meta-analytic decisions such as dealing with multiple effect sizes, or the eligibility criteria used by each study. The coding form for this study is available at https://osf.io/6d8be/. Data was extracted and coded independently by two authors (ASL and MT), except for the information regarding preregistered meta-analyses comparing the protocol and the final articles. Discrepancies were solved by consensus. For each included article, we extracted information about: basic characteristics of the article (journal, publication year, country, trained domains, population, funding sources, and conflicts of interest), preregistration, adherence to reporting guidelines, search and inclusion of the studies (information sources, search strategy, eligibility criteria, selection process), data extraction procedure, effect size measures, meta-analytical methods (statistical model, weights, between-studies variance estimators, combining multiple effect sizes, and software), assessment of the quality of primary studies, inspection of publication bias, and data and script availability.

In each meta-analysis, we coded any reported information about each of those stages, distinguishing when they reported that they did or did not perform a specific step. When a meta-analysis reported that they performed that step, we coded the specific procedure to examine which were the most frequent practices. For example, if authors explicitly reported that they did inspect publication bias, we coded “reported, they inspected publication bias” and coded which methods for inspecting publication bias were used. However, if they explicitly stated that they did not inspect publication bias, we coded “reported, they did *not* inspect publication bias.”

In the cases where the article reported having a preregistered protocol, we examined the protocols to assess their coverage of key meta-analytic decisions and possible deviations from the protocol. The coding scheme is adapted from https://osf.io/kf9dz, and we evaluated the following aspects: eligibility criteria, outcomes, outcome prioritization, risk of bias, meta-analysis model, dealing with multiple effect sizes, additional analyses, and publication bias. Three types of deviations were considered: (a) if there is information regarding some decision in the protocol but this information is not present in the final article, (b) if there is information regarding some decision reported in the final article but this was not pre-specified in the protocol, and (c) if there is information in both the protocol and the final article but there is different information. Besides, we also coded whether deviations were disclosed or not.

### Data Analysis

All analyses were performed using R Statistical Software (v4.1.2; R Core Team, 2021). For each item, we calculated the proportion of meta-analyses in which the information was available. For this, we used the R package “*DescTools*” (Signorell et al., 2019). We also examined the possible association between publication year and the reporting of information using binary logistic regression, to see whether report trends have change over the years, for every item that was reported less than 80% of the time. For the data preparation and presentation, we used the packages included in “*tidyverse*” (Wickham et al., 2019). Scripts and data are available at https://osf.io/jw6rb/.

## Results

Our search retrieved a total of 1983 results, which, after the removal of duplicates, yielded a total of 919 results. Of these, 659 studies were screened by title or abstract. Subsequently, we assessed 260 full-text articles and included 87 articles. Additional searches scanning the references retrieved 10 additional eligible articles, leaving us with a total of 97 meta-analytic studies. Most of these 97 meta-analyses selected in this meta-review included primary studies that applied a training program targeting several cognitive domains (63; 65%) or exclusively working memory (20; 21%). The rest of studies focused on executive functions, memory, attention, or spatial skills. There was substantial diversity in the populations examined across different meta-analyses, which assessed the benefits of cognitive training in a wide range of clinical conditions such as schizophrenia, multiple sclerosis or mild cognitive impairment considering any age, from children to older adults (see Supplementary Table 2 at https://osf.io/j58ax/ for the characteristics of the included studies). Meta-analyses were published between 1997 and 2023 (median = 2019), with 40 being published in 2020 or afterwards (see Supplementary Fig. 2 at https://osf.io/mp8ye/).

### Preregistration, Guidelines, Conflict of Interest, Funding Sources, and Data Availability Statement

**Fig. 1:**
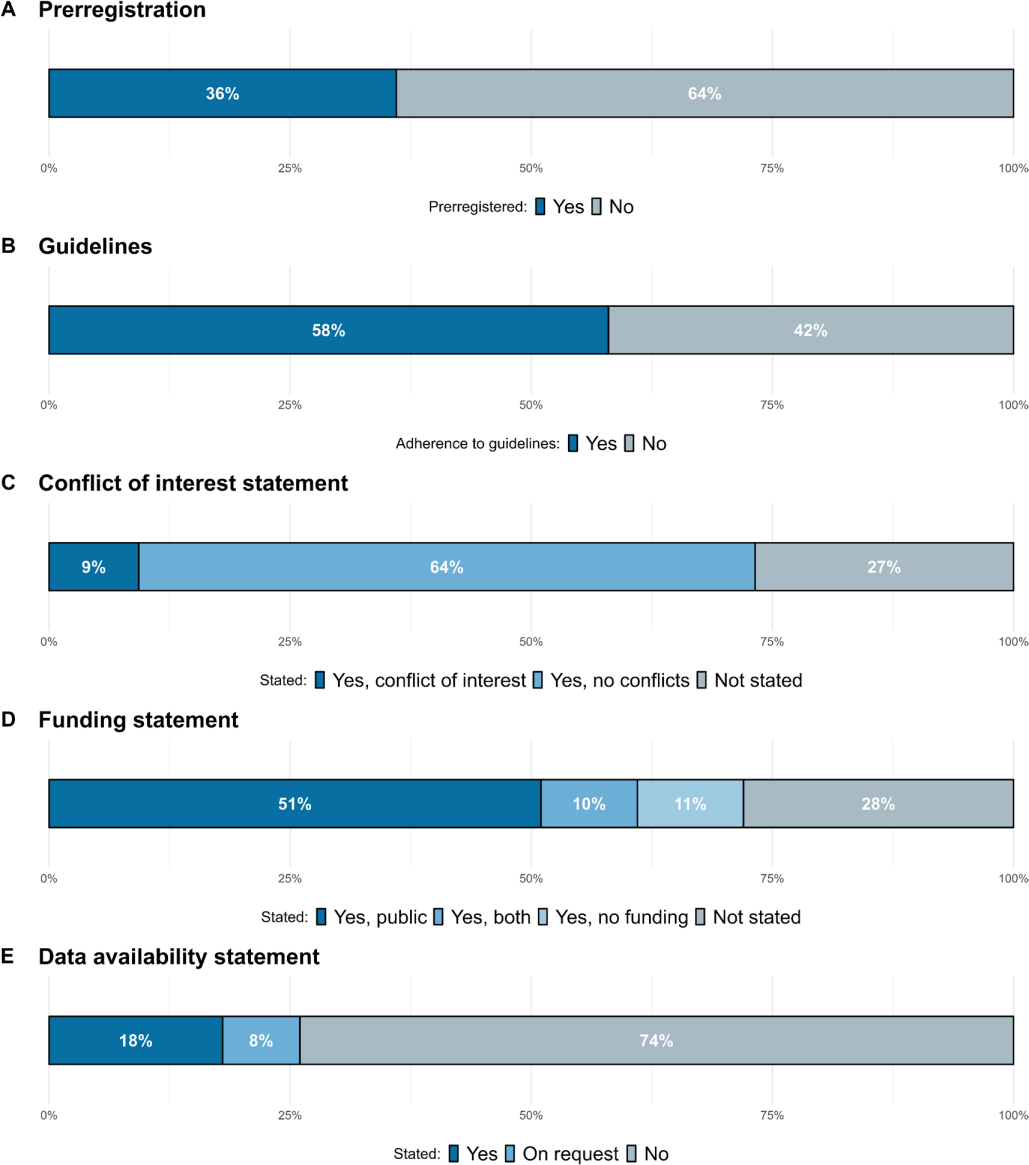
Percentage of meta-analyses that reported being preregistered (**A**), following reporting guidelines (**B**), having a conflict of interest (**C**), their funding (**D**), and a data availability statement

Of the 97 meta-analyses included in our meta-review, 35 (36%)^1^ reported having preregistered a protocol (see Fig. 1a), with 30 (86%) using PROSPERO, 3 (9%) having their protocol published in the Cochrane Database of Systematic Reviews, 1 (3%) using INPLASY, and 1 (3%) sharing it in the supplementary materials. A total of 56 studies (58%) reported using any guidelines or checklists for the reporting of meta-analysis information (see Fig. 1b), where 53 (95%) used the PRISMA guidelines, 1 (2%) used the MARS, 1 (2%) used Cochrane guidelines, and 1 (2%) used APA guidelines. In total, 9 (9.3%) studies disclosed some conflict of interest, 62 (63.9%) reported having no conflict of interest, and 26 (26.8%) did not include a conflict of interest statement (see Fig. 1c). Finally, 70 (72%) studies included a funding statement, with 49 (51%) having only public funding, 10 (10%) having both public and private funding, and 11 (1%) reporting having no funding, while 27 (28%) did not include any information about funding sources (see Fig. 1d). Lastly, only 25 (26%) of the studies included a statement regarding the availability of the data, with 8 (8%) of them specifying that data would be available on request.

### Eligibility Criteria, Information Sources, Search Strategy, and Selection Process

**Fig. 2:**
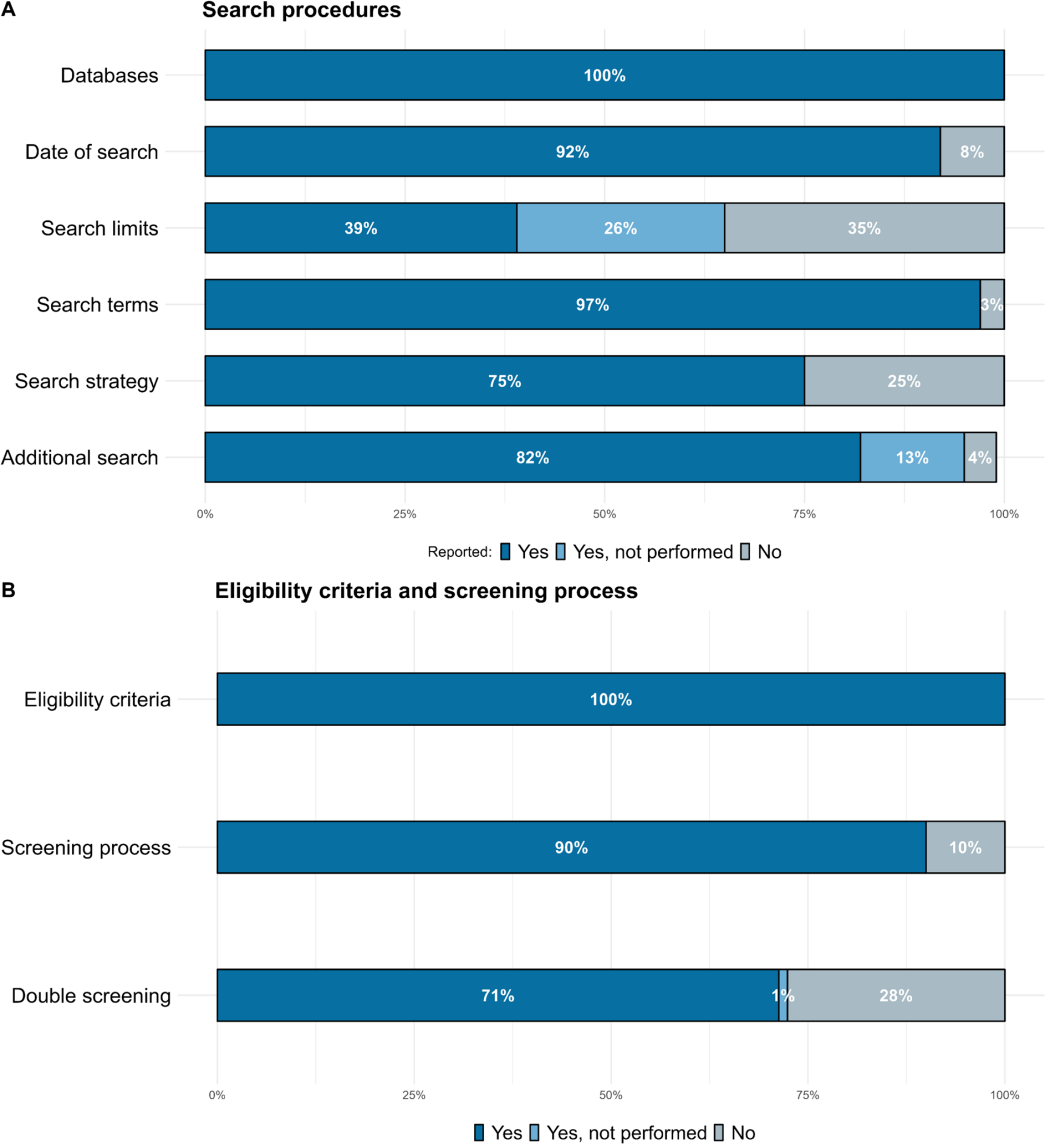
Percentage of meta-analyses that reported information about the search procedures (**A**) and eligibility criteria and screening process (**B**)

All 97 meta-analyses reported the electronic databases consulted (see Fig. 2A). 89 (92%) studies reported the date when the electronic search was conducted. Furthermore, 80 (82%) reported using additional search strategies, and 13 (13%) reported not using any other strategy. The most frequently used method to conduct an additional search was to consult the references of the articles was the preferred method (60 studies; 84%). 38 studies (39%) indicated imposing some search limits, 25 (26%) reported not setting any search limit, and 34 (35%) did not report any information regarding search limits. 94 studies (97%) reported the search terms that were used, although 73 (75%) reported the exact search strategy for, at least, one database. All the studies reported information about the eligibility criteria (See Fig. 2B). Inspecting the eligibility criteria using the PICO scheme showed that meta-analytic studies used very different criteria to decide which primary studies could be eligible. The largest variation was found for the training duration, where meta-analyses required the following durations: at least 4 h (Hallock et al., 2016; Hill et al., 2017; Lampit et al., 2014, 2019; Leung et al., 2015), more than 1 session (das Nair et al., 2016; Loetscher et al., 2019; Melby-Lervåg et al., 2016; Weicker et al., 2016), at least 3 sessions (Nguyen et al., 2019), at least 10 sessions (Giustiniani et al., 2022; Scionti et al., 2020), at least 20 sessions (Spencer-Smith & Klingberg, 2015), at least 1 week (Au et al., 2015), at least 2 weeks (Schwaighofer et al., 2015), from 4 to 12 weeks (Dardiotis et al., 2018), at least 12 weeks (Gates et al., 2020), and at least 1 month (Bonnechère et al., 2020). In contrast, 79 studies did not specify any required duration. Additionally, several cut-offs were used for the different age ranges. For example, studies that focused on children and adolescents often considered different age ranges such as 3 to 12 years (Cao et al., 2020; Kassai et al., 2019), 3 to 18 years (Chen et al., 2021; Cortese et al., 2015), 6 to 19 years (Oldrati et al., 2020), less than 18 years (He et al., 2022), less than 19 years (Robinson et al., 2014), and less than 21 years (Corti et al., 2019). Four studies did not specify their cut-offs. Similarly, when considering only older adults (25 studies), meta-analyses often required participants to be 60 years old or older. However, 3 (Basak et al., 2020; Ha & Park, 2023; Kelly et al., 2014) and 2 (Tetlow & Edwards, 2017; Zhang et al., 2019) studies also used 50 and 55, respectively, as the age cut-off. Variability was also found regarding the design of the primary studies, where 54 (56%) meta-analyses required primary studies to be randomized controlled trial (RCT), 33 (34%) studies allowed non-RCTs, and 10 (10%) did not report any information. Similarly, 39 (40%) studies included gray literature, 29 (30%) excluded it, and 29 (30%) did not report any information about including or excluding this literature. 16 (16%) studies reported allowing another intervention to be delivered in combination with cognitive training, with 6 (5%) studies (Aksayli et al., 2019; Goldberg et al., 2023; Hallock et al., 2016; Lampit et al., 2014, 2019; Leung et al., 2015) specifying that cognitive training had to conform 50% of the intervention and 1 (1%) study (McGurk et al., 2007) requiring that cognitive training was the 75% of the intervention. Last, 9 studies did not require that the entire sample of a study was their target population in order to include the study, again using different cut-offs: 50% (Tetlow & Edwards, 2017; Woolf et al., 2022), 60% (Goldberg et al., 2023), 70% (Kambeitz-Ilankovic et al., 2019; Wykes et al., 2011), 75% (das Nair et al., 2016; McGurk et al., 2007), and 80% (Gates et al., 2020; Yan et al., 2023).

Finally, most studies, 87 (90%), detailed the screening process (i.e., how many articles were retrieved and how many were screened by title or abstract), with 62 (71%) reporting that a double screening was performed, and 1 (1%) acknowledging that it was conducted by a single author. However, not every article that described their screening process specified in detail the reasons for screening the articles. The majority specified the number of articles that were for excluded for each reason, with only 11 (13%) specifying the exact reason for each article that was considered not eligible.

### Data Collection Process

**Fig. 3:**
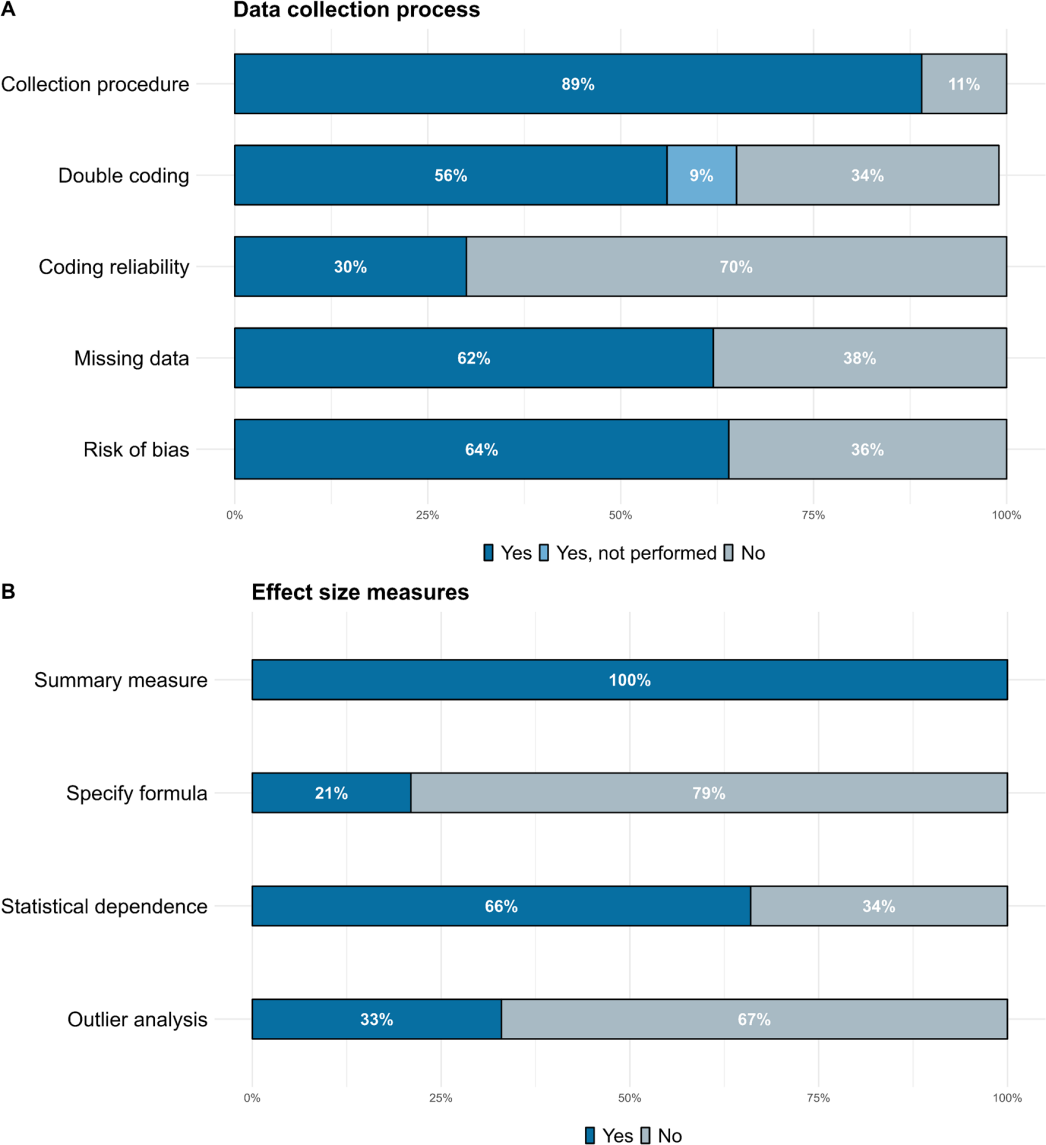
Percentage of meta-analyses that reported information about the data collection process (**A**) and effect size measures (**B**)

Most meta-analyses, 86 (89%), reported information about the data collection procedure (see Fig. 3A). Of these, 54 (56%) reported double coding the information, with 16 (30%) reporting some measure of inter-rater reliability, and 9 meta-analyses (9%) detailed that the coding was not performed by two authors. 60 studies (62%) reported using some procedure to deal with missing data, mainly by reaching the original authors (54 studies; 92%). 62 studies (64%) reported assessing the risk of bias of individual studies procedure (see Fig. 3A).

### Effect Size Measures, Statistical Dependency, and Outlier Analyses

All meta-analyses reported which effect size measure they used in the synthesis (see Fig. 3B). However, only 20 (21%) specified the exact formula to compute the summary measure. Besides, 27 (28%) studies did not specify the exact standardized mean difference (SMD) measure that was used. Of the studies that specified this information, the majority, 44 (63%), reported using a pre-post controlled SMD, which was followed by using a post-test SMD (20 studies; 28%). The rest of the studies (6 studies, 9%) used a pre-post SMD or several measures. Of the studies that used a pre-post controlled SMD, only 4 studies specified which correlations they used for calculating the sampling variance and another 4 specified that they used a formula that did not require to input any correlation. On the other hand, 64 studies (66%) reported some method to deal with statistically dependent effect sizes. The most popular choice, reported by 37 studies (58%), was averaging multiple effect sizes. Selecting only one measure applying some decision rule was used in 10 studies (16%). 3 studies (5%) used both averaging and following a decision. 13 (20%) studies opted for modeling the statistical dependency, where robust variance estimation was the preferred method with 7 studies (11%), followed by multi-level meta-analysis (6 studies; 9%). One study used the Cheung and Chan method to merge statistically dependent effect sizes (See supplementary Table 3 at https://osf.io/j58ax/ for more details). Lastly, 32 studies (33%) reported conducting outlier analyses.

### Meta-analytical Methods

**Fig. 4:**
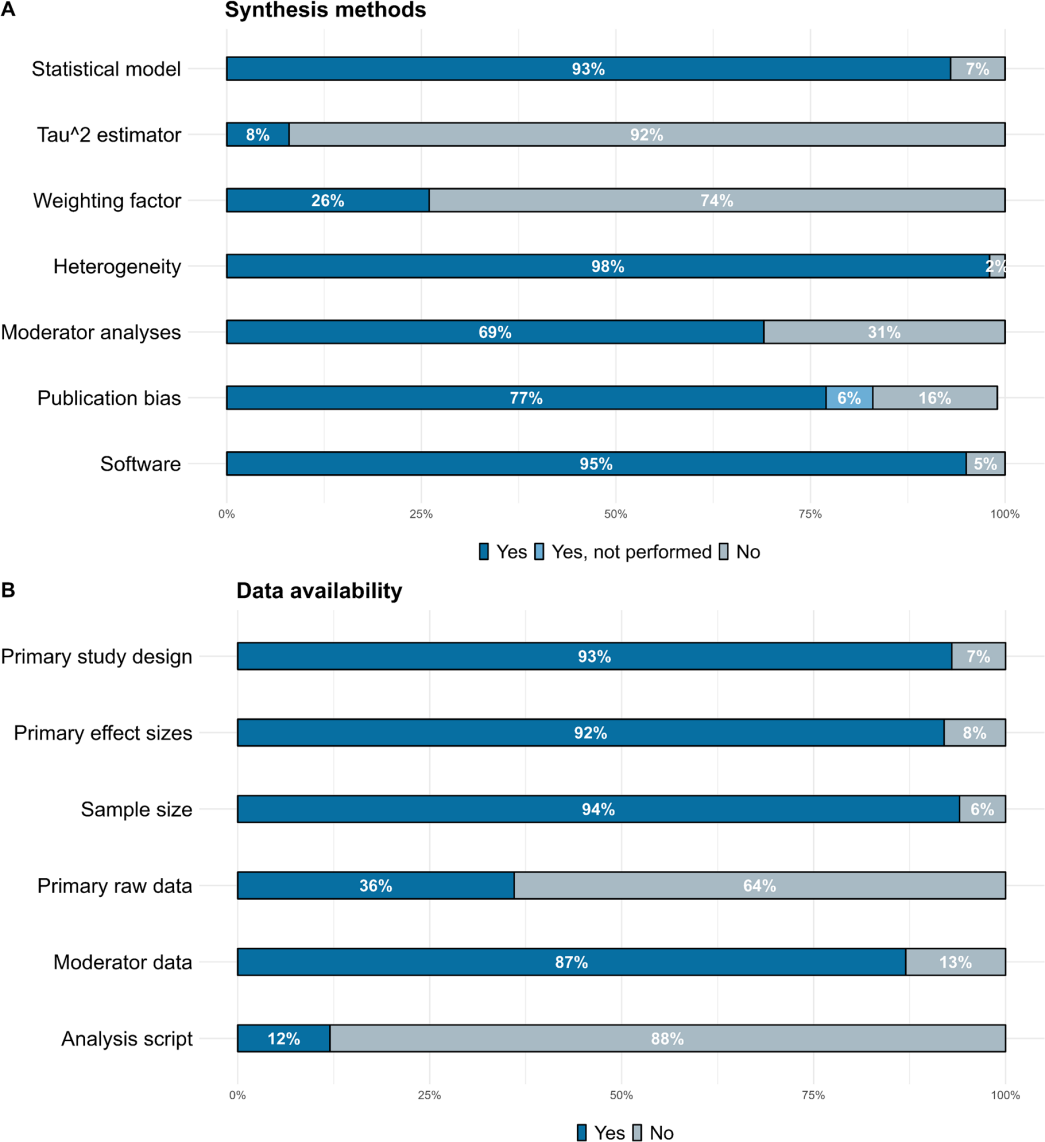
Percentage of meta-analyses that reported information about synthesis methods (**A**) and data (**B**)

Most meta-analyses stated the statistical model assumed for the synthesis (90 studies; 93%) (see Fig. 4A). Of these 90 studies, the most frequently used model, used by 62 studies, was the random-effects model. 12 studies used both random-effects models and fixed-effect models, according to the heterogeneity. Only 3 used a fixed-effect model uniquely. 13 studies used models to account for statistically dependent effect sizes. 25 studies (26%) specified the weighting scheme. Besides, of those studies that did not assume a fixed-effect model, only 8 reported the between-studies variance estimator. 95 meta-analyses (98%) reported having assessed heterogeneity. 67 (69%) reported moderator analyses. 75 (77%) reported publication bias assessments, while 6 (6%) explicitly reported not performing them. There was diversity in the methods that were used to inspect publication bias, with most studies using multiple methods (54 studies; 72%). The most popular methods were funnel plots (54 studies; 85%), Egger’s regression (28 studies; 52%), Trim-and-Fill (22 studies; 41%), and Fail-Safe N (14 studies; 26%) (See Supplementary Tables 4–5 at https://osf.io/j58ax/ for more details). Only 8 (11%) studies used methods other than those mentioned above. Of the studies that inspected publication bias, 6 (8%) found substantial evidence of potential publication bias, with half of them attempting to correct the effect sizes. 20 (27%) studies found some evidence for the presence of publication bias in, at least, one outcome with, again, half of them attempting to correct for this. 4 (5%) studies did not discuss the presence of publication bias despite reporting that publication bias was inspected. Finally, 92 (95%) reported the software, where “Comprehensive Meta Analysis” (34 studies; 38%) and “RevMan” (27 studies, 30%) were the most popular choices, followed by an *R* package (21 studies; 23%).

### Data Availability

Most meta-analytical studies (90 studies; 93%) reported information about the characteristics of the primary studies that were included in the synthesis. 89 (92%) reported the effect sizes of the primary studies. Of these 89 studies, 73 (82%) made this information available only in a .PDF or .DOC format, with only 11 (12%) sharing the data files in .CSV or Excel files, and 5 studies (6%) sharing it through a RevMan file (See Supplementary Table 6 at https://osf.io/j58ax/). Only 34 (36%) of the studies reported the primary raw data (means and standard deviations of the primary studies) that they used to compute the effect sizes. Of these 34 studies, 22 (64%) reported this information in a .PDF or .DOC format, only 6 (18%) shared the data files in .CSV or Excel files, and 6 (18%) studies reported it through a RevMan file (See Supplementary Table 7 at https://osf.io/j58ax/). 88 studies (94%) reported the sample sizes of the primary studies, with 76 studies (86%) reporting this information in .PDF or .DOC format (See Supplementary Table 8 at https://osf.io/j58ax/). Most studies (57 studies; 87%) that performed a moderator analysis reported the data of the moderating variables, with 51 studies (89%) reporting this information in .PDF or .DOC format (see Supplementary Table 9 at https://osf.io/j58ax/). Finally, the analysis script, such as the R code or the SPSS syntax, was only available for 12 studies (12%).

### Preregistered Meta-analyses

**Fig. 5:**
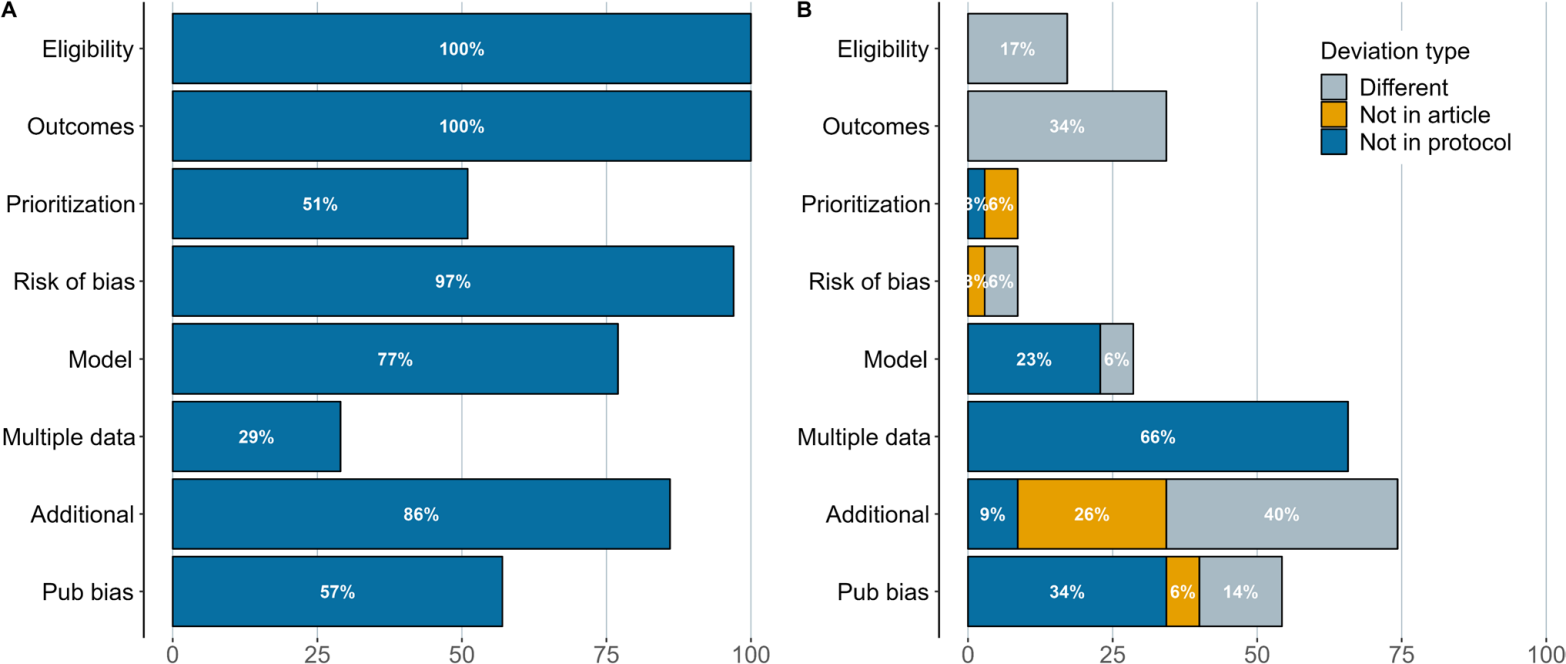
Percentage of preregistered meta-analyses that specified information about each aspect in their protocol (**A**) and percentage of preregistered meta-analyses that contained deviations (**B**)

Most of the 35 preregistered meta-analyses pre-specified information regarding the eligibility criteria, outcomes of interest, risk of bias assessments, statistical model, and additional analyses in their protocols (see Fig. 5 for details). However, only 10 (29%) studies detailed in advance how they would deal with the multiplicity of effect sizes and 20 (57%) how they would inspect publication bias. Information regarding these aspects was later specified in the final article in most cases, which accounts for the 66% and 34% of deviations due to information being in the final article but not in the protocol for the items regarding multiplicity of data and publication bias, respectively. Besides, the largest deviations due to information being present in both protocol and final article but with differences were found in the eligibility criteria (6; 17%), outcomes of interest (12; 34%), additional analyses (14; 40%), and publication bias (5; 14%). Finally, some other deviations were found because the information was specified in the protocol but not in the final article. This was found in the prioritization of outcomes (6%), risk of bias (3%), additional analyses (26%) and publication bias (6%). Of note, deviations were rarely disclosed (See Supplementary Fig. 3 at https://osf.io/mp8ye/). Deviations were only mentioned in 17% of the cases regarding the outcomes of interest, 38% of the cases regarding additional analyses, and 11% of the cases regarding the inspection publication bias. None of the rest of the deviations were disclosed.

### Association Between Publication Year and Transparency

The following variables were inspected: preregistration, following guidelines, setting search limits, providing the search strategy, performing double screening and coding, calculating the reliability of the coding, assessing risk of bias, providing effect size formulae, modeling statistically dependent effect sizes, inspecting outliers, reporting the between-studies variance estimator and weighting scheme, performing moderator analyses, inspecting publication bias, inspecting publication bias with methods other than the Funnel Plot/Egger’s regression/Trim and Fill, reporting the raw data (means, SDs, and Ns), using R as the meta-analytic software, sharing the analysis script and stating whether the data is available. Supplementary Table 10 (https://osf.io/j58ax/) shows the results for all the variables that were explored. We found statistically significant associations (*p* < 0.05) between year and the following variables: preregistration,^2^ following guidelines, setting search limits, reporting the search strategy, risk of bias assessment, inspecting outliers, weighting scheme, inspecting publication bias, using R, and reporting the raw data. On the contrary, reporting a double screening or double coding, detailing the formulae, modeling statistically dependent effect sizes, reporting the between-studies variance, performing moderator analyses, inspecting publication bias with other methods, sharing the analysis script, and stating whether data is available did not seem to change over the years.

## Discussion

Meta-analytical studies require many decisions regarding the inclusion of studies, analysis, or evaluation of the primary studies. Thus, they present a flexibility equal or greater than empirical studies. Considering how relevant meta-analyses are as well as the controversial results found in the field of cognitive training, it is of great importance that meta-analyses on this topic report transparently every decision that could influence the results or the conclusions. In this study, we examined the transparency practices in the meta-analyses of cognitive training published up to July 2023. Moreover, in contrast to previous studies examining the reporting of information in meta-analyses (López-Nicolás et al., 2022; Polanin et al., 2020) or meta-reviews related to cognitive training (Gavelin et al., 2020), we provide a comprehensive overview of the methods that these meta-analytic studies used, which allowed a critical evaluation of these practices. Besides, we provide information regarding the eligibility criteria that the meta-analyses have used, and we also inspected the preregistered meta-analyses and compared them with their final article.

We found 97 meta-analytical studies to be eligible for our meta-review, spanning more than 20 years. These studies covered a wide range of populations, including people with very varied clinical conditions and age ranges, training interventions with diverse delivery methods and target cognitive domains, and multiple measured outcomes. Similar to what is found in Gavelin et al. (2020), more than one third of the meta-analyses had a preregistered protocol, a higher prevalence than what was found in previous meta-reviews from other fields (López-Nicolás et al., 2022; Polanin et al., 2020), and much higher than what is typically found for primary studies (Hardwicke et al., 2020). It is also worth mentioning that some of the meta-analyses were published at a time when there was not even a platform for their preregistration, since PROSPERO was launched in 2011 (Page et al., 2018). Importantly, even if we only consider the studies that were published after its launch, the practice of preregistering meta-analyses seems to be increasing over the years. Nonetheless, inspecting the protocols of these preregistered meta-analyses revealed important issues. Authors often failed to pre-specify key meta-analytic decisions such as how they would deal with the multiplicity of data or inspect publication bias. Besides, we found changes between the protocol and the final article regarding critical aspects such as the eligibility criteria or the outcomes of interest. We also found cases where there was information specified in the protocol, but they did not report anything in the final article in items such as the prioritization of outcomes or additional analyses. Equally important, these deviations were rarely disclosed. Although these issues also have been found in other empirical studies and systematic reviews (Bakker et al., 2020; Claesen et al., 2021; Tricco et al., 2016; van den Akker et al., 2023), the risk of bias arising from these deviations can be especially sensitive given the state of the field.

We found modest rates of following guidelines for conducting and reporting a systematic review such as the PRISMA. Nonetheless, this practice has increased over the years and is also slightly higher than what previous studies (López-Nicolás et al., 2022) have found. However, it is also worth noticing that reporting following such guidelines does not guarantee reporting every aspect included in the guidelines. On the other hand, about one third of the studies did not report whether they had any conflict of interest or whether they received any funding. Although we acknowledge that this information is not always required by the journals, we recommend that this information be included considering that cognitive training is, in many cases, used with financial interests. As previous studies have shown, presenting conflicts of interests or receiving private funding can bias the results of systematic reviews and meta-analyses (Cristea et al., 2017; Ebrahim et al., 2016; Ferrell et al., 2022; Yank et al., 2007).

The best reporting practices were found regarding the search and screening procedures, since most studies reported aspects such as the electronic databases, the date of the searches, the exact search strategy, and additional search methods. Besides, all of them reported the inclusion criteria. Nonetheless, a careful inspection of the inclusion criteria revealed substantial heterogeneity. In aspects such as the required duration training, studies used up to 10 different duration criteria, with many studies not specifying any required duration. There was also wide diversity in the cut-offs used for the different age ranges, where studies also used up to 6 different criteria when their target population was children. Likewise, authors usually considered different requirements for study designs, publication status or interventions that could be applied in combination with cognitive training. Acknowledging these differences is essential because they have, intentionally or not, an impact on the results of the meta-analysis.

Another aspect to highlight is that over one-third of the studies of the did not provide an assessment of the risk of bias of the primary studies, akin to what was found in Gavelin et al. (2020). Although this practice seems to have improved over the years, this prevalence is lower than what is found in previous studies (López-Nicolás et al., 2022). Evaluating the risk of bias of primary studies is critical because the evidence provided by the meta-analyses depends on the quality of the primary studies (Kvarven et al., 2020; Savović et al., 2012) and cognitive training primary studies usually do not adhere to best-practice standards (Gobet & Sala, 2022; Sala & Gobet, 2019; Simons et al., 2016). Importantly, while meta-analyses cannot remedy the problems of primary studies, they should provide a transparent and comprehensive assessment of the strength of the primary studies, as it is the only way that allows for a thorough evaluation of the degree of confidence we can have in the results of a meta-analysis.

All meta-analyses reported the effect size measure used. However, some of them did not detail the exact SMD measure and few of them reported the specific formula, a practice that has not changed over the years. Besides, some studies that reported using a pre-post controlled SMD did not specify the imputed correlation. These aspects are also important to detail as different computations for the different effect size measures are available, and the choice may affect the synthesized results (López-Nicolás et al., 2022; Rubio-Aparicio et al., 2018). On the other hand, the majority of studies reported addressing the statistical dependency that arose from the multiplicity of effect sizes, although this was mainly accomplished by averaging dependent effect sizes or only selecting one effect size, which leads to loss of information (López-López et al., 2018). Importantly, this practice has not changed over the years. Future studies should consider accounting for dependent effect sizes by using models with robust variance estimation (RVE; Pustejovsky & Tipton, 2022; Tanner-Smith et al., 2016) or multilevel meta-analyses in order to make use of all the available information (Gucciardi et al., 2022; López-López et al., 2018; Van den Noortgate et al., 2015).

Regarding the synthesis methods, it was very common to report the statistical model assumed, assessing heterogeneity, conducting moderator analyses, and specifying the meta-analysis software. However, most meta-analyses failed to specify other relevant details such as the between-study variance estimator and the weighting scheme, which can also affect the results (Veroniki et al., 2016). Studies frequently inspected the possibility of publication bias in their results, which also was an increasingly popular practice. Of note, about one third found some evidence of publication bias in at least one outcome, with some stating that the potential for publication bias was substantial. The most frequent methods were inspecting funnel plots, using Trim-and-Fill, Egger’s regression or even the Fail-Safe N. Importantly, while it is true that earlier studies did not have such a variety of methods available, our analyses show that more recent meta-analytic studies are not applying other more modern techniques frequently. Future studies might consider alternate methods depending on the characteristics of each meta-analysis (Maier et al., 2022; van Aert et al., 2016), since the popular approaches mentioned above might not be the optimal in many applied scenarios.

Finally, regarding data availability, we found good reporting rates of the primary study design, effect sizes, sample sizes, and moderator data. Similar to previous studies examining data sharing practices (Polanin et al., 2020 and López-Nicolás et al., 2022), the worst rates were found for sharing raw data (means and SDs), which prevents recalculating the effect sizes, and sharing the analysis script. Besides, it is noteworthy that, although this practice seems to be improving over the years, most of the data that was available was reported in non-machine-readable formats such as .PDF or .DOC files, which requires to manually re-code data in case anyone wanted to reuse the data for further re-analyses or make reproducibility checks. Manually re-coding data is an especially time-consuming and error-prone task, so sharing data in a machine-readable format such as .CSV is preferred.

### Recommendations for Future Cognitive Training and Other Cognition-Oriented Meta-analyses

A pragmatic way to ensure the correct reporting of information is to follow established reporting guidelines such as the PRISMA, which exhaustively covers meta-analytic aspects, from the search strategy to the conflicts of interest and funding. Besides, we strongly encourage sharing the data to allow other authors to reproduce and make re-analyses using different methods in machine-readable formats such as .CSV. Note that, because the primary unit of analysis is summary data, which has already been shared publicly, sharing meta-analytic data does not entail any ethical concern. Therefore, there is no good reason to not share the data (López-Nicolás et al., 2022; Moreau & Gamble, 2022).

On the other hand, being completely transparent lies beyond simply reporting the information about the methods. Providing a careful rationale for each decision as well as acknowledging the possible alternatives is essential, especially regarding eligibility criteria, which is, arguably, one of the most important decisions (Harrer et al., 2021; Voracek et al., 2019). Besides, when several decisions are possible, performing sensitivity analyses can be desirable.

Moreover, a transparent reporting of all the decisions and data is necessary, but not sufficient, to ensure the quality of a meta-analytical study. A completely transparent study can still suffer from outcome-dependent biases, poor methodology, or biases arising from the primary studies. A practical solution that minimizes the possibility of outcome-dependent biases and is often regarded as a best practice is to preregister a study protocol detailing the research questions, inclusion criteria and planned analyses prior the beginning of the study (Hardwicke & Wagenmakers, 2021; Moreau & Gamble, 2022; Nosek et al., 2019; Pigott & Polanin, 2020). Nonetheless, protocols should be comprehensive, specifying all key meta-analytic decisions. In this context, deviations from the protocol are natural and sometimes even desired, as long as they are disclosed and discussed. These points are essential since the effectiveness of the preregistration depends mainly on these aspects.

On the other hand, the evidence provided by a meta-analysis is just as good as the primary studies that are included in the analyses. It is well known that primary studies that do not comply with aspects such as a correct randomization can introduce bias to the results, and it has been shown that primary studies from the cognitive training field usually suffer from those important shortcomings (Savović et al., 2012; Simons et al., 2016). For this reason, it is very important that future reviews evaluate the risk of bias and discuss the evidence considering the results of this assessment. Finally, addressing methodological aspects, it is common for cognitive training studies to report multiple effect sizes even from the same cognitive domain. Accounting for statistically dependent effect sizes in your model might be preferred over averaging effect sizes or selecting only one effect size according to some decision rule. We refer to the work of Cheung (2019), López-López et al. (2018), and Moeyaert et al. (2017) for a detailed review and guidelines of this topic. Lastly, regarding the assessment of publication bias, it is important to note that not all methods to inspect publication bias work well under conditions such as small number of primary studies or high heterogeneity. Therefore, authors must be cautious selecting the analyses and consider using alternative methods (Maier et al., 2022) or a combination of them (Siegel et al., 2021; van Aert et al., 2019) to provide a more nuanced evaluation taking into account their specific situation.

### Limitations of the Current Study

Our study presents some limitations. First, this work is purely descriptive and only focuses on the reporting practices of the cognitive training meta-analyses. We discarded conducting analyses examining the relationship between the reporting and the results of the meta-analyses because meta-analytic studies often synthesized several outcomes, with both significant and non-significant results. Therefore, trying to relate the reporting quality with the results of meta-analyses reporting both significant and non-significant effect sizes can be very challenging. Besides, it is important to acknowledge that reporting or not reporting information about an aspect does not necessarily have to be related with the results, in any specific direction. For this reason, no conclusions about the actual benefits of cognitive training can be drawn from the results presented here. Future studies might want to examine the relationship between the reported methodological practices and the results by conducting re-analyses of the meta-analyses comparing different methods and checking whether the conclusions change.

Another point to consider is that, as we mention above, the issue of transparency lies beyond simply reporting the information of each aspect and requires providing a rationale for each decision. Unfortunately, although we attempted to examine whether studies provided a justification for the meta-analytic decisions, we were unable to examine this issue for several reasons. First, considering adding a citation or some kind of argumentation as providing a rationale posed some problems, because, although some studies provided a justification of this kind, their adequateness was debatable. See, for example, recent studies included in our meta-review citing older methods for aspects such as how to deal with the multiplicity of effect sizes or publication bias. Therefore, we think that treating these cases as providing a rationale can be misleading. Second, when considering citations, evaluating whether studies provided a rationale not only requires checking whether authors provide a citation to back up their decision, but also requires examining that the citation actually supports the assertions. In fact, we found cases where it does not. For example, Ha and Park (2023) reported that the cut-off age for studying older adults was set at 50 years old, following guidelines provided by Shenkin et al. (2017) for studying older adults. However, what Shenkin et al. (2017) discuss is that, although there is no generally agreed criterion to define “older people,” a cut-off of over 60 or 65 years is often used. For all this, a careful examination of this issue would require an amount of work that is out of the scope of the present review.

Finally, it is worth mentioning that many reviews can be found which examine the benefits of cognitive training combined with additional interventions such as physical exercise or non-invasive brain stimulation. Although excluding these studies reduced potential heterogeneity between studies, making them more comparable, examining them could also offer interesting information. However, due to our resource constraints, this was not feasible in the current study. Similarly, for this reason, the screening process and the comparison between preregistered protocol and final articles was conducted by a sole author.

## Conclusions

We found that the reporting of information and data of cognitive training meta-analyses was transparent in aspects such as how they performed the search of the studies, the screening procedure, or the data collection, akin to what previous studies examining other disciplines have found. Additionally, many reporting practices seem to have improved over the years. However, we found that the reporting quality was poorer in aspects such as specifying the exact meta-analytic parameters, formulas or sharing the data. Besides, other aspects such as the methods to deal with multiple effect sizes from the same study, analyses to assess the presence of publication bias and some data-sharing practices did not change over the years. Moreover, we found a remarkable heterogeneity in the eligibility criteria that needs to be better addressed in future studies. Also, we observed that meta-analyses that were preregistered often did not specify important decisions, and some contained non-disclosed deviations. Even though these results cannot be directly related with the results of the meta-analytic studies, they illustrate important points that must be addressed in future studies, especially considering how disputed the benefits of cognitive training programs are at present. In this context, we emphasize the need for a complete and detailed reporting of every decision that could influence the results, providing a clear and thoughtful rationale for each decision and, ideally, sharing all the meta-analytical data to allow any reproducibility check or further re-analyses incorporating other methods that might be more suitable.

## Data Availability

The datasets analyzed during the current study and the script code for generating processed data tables and performing statistical analyses are available in the Open Science Framework repository (https://osf.io/jw6rb/).

## Appendix. Decisions to Be Made in a Cognitive Training Meta-analysis

This is a non-exhaustive scheme of the possible decisions to be made before conducting a meta-analysis which can have a direct impact on the results and conclusions. The following list includes common items in systematic reviews and meta-analyses that can be found in the PRISMA 2020 checklist (Page et al., 2021):

1. What should be the target population? We could include studies that apply cognitive training to any kind of population, but also, we could select only those studies that apply it to people with a particular clinical condition (e.g., patients with an acquired brain injury). We could also select studies with participants of any age or limit it to a particular range of years (e.g., only older adults).
2. Which kind of cognitive training intervention should be included? We could include studies that use a multi-domain training intervention, studies that only train a particular domain (e.g., working memory), or studies that only deliver training under certain formats such as computerized or commercial programs (e.g., Cogmed). Besides, we could allow or exclude using other treatments in combination such as physical interventions or non-invasive brain stimulation.
3. Which outcomes should be considered? Authors in this field often measure a wide range of outcomes. We could include all possible outcomes or focus only on a subset of them (e.g., checking the impact only on measures of intelligence or daily functioning).
4. Should we impose any further restrictions to the studies that we include? We could include studies from the “gray literature” (e. g., unpublished studies), and we could also limit the publication year in which studies were published (e.g., including only trials from 1990 onwards).
5. Which summary measure should be used? Given the nature of the studies, a summary measure based on standardized mean differences (SMD) might be appropriate. However, depending on the study design there are different indices available, each one with different possible computations (Rubio-Aparicio et al., 2018).
6. How should we deal with multiplicity of effect sizes? As stated before, it is frequent that the same study reports effect sizes for several cognitive domains and even multiple effect sizes for a single cognitive domain. This multiplicity of effect sizes can result in statistically dependent effect sizes, and there are several ways to handle them (Cheung, 2019; López-López et al., 2018; Moeyaert et al., 2017). Common approaches include running independent meta-analyses, merging dependent effect sizes, or only selecting one outcome per study, but we could also model that dependency using Robust Variance Estimation (RVE), a three-level meta-analysis or a multivariate meta-analysis (Cheung, 2019; Hedges et al., 2010; Moeyaert et al., 2017; Van den Noortgate et al., 2015).
7. Which meta-analytical model, between-study variance estimator, and weighting factors should be used? Currently, there are many options beyond the traditional, univariate fixed-effect/random-effects meta-analyses, such as network meta-analyses, Bayesian meta-analyses, or meta‐analytic structural equation modeling (MASEM), to name a few. Additionally, there are many different between-study variance estimators available, and there are also different ways to weigh each study (López-Nicolás et al., 2022; Veroniki et al., 2016).
8. Last, should additional analyses be conducted to examine small-study bias or publication bias? Again, many different tools are available at this stage (Siegel et al., 2021; van Aert et al., 2019); from the classic funnel plots (Light & Pillemer, 1984) and Egger’s regression test (Egger et al., 1997) to more recent techniques such as PET-PEESE (Stanley & Doucouliagos, 2014), selection models (Vevea & Woods, 2005), p-curve (Simonsohn et al., 2014), p-uniform (van Aert et al., 2016), or RoBMA (Bartoš et al., 2022).

## Footnotes

1 PROSPERO, the leading platform for preregistering systematic reviews and meta-analyses, was launched in 2011 (Page et al., 2018). If we consider only studies that were conducted after its implementation, the prevalence of preregistration increases to 38%.

2 A sensitivity analysis that included only studies published after the launch of PROSPERO in 2011 also showed a significant association between year and preregistration.
